# Squamous Cell Papilloma of the Esophagus: A Case Series Highlighting Endoscopic and Histologic Features

**DOI:** 10.1155/2020/7645926

**Published:** 2020-06-01

**Authors:** Dustin J. Uhlenhopp, Kristin M. Olson, Tagore Sunkara

**Affiliations:** ^1^Department of Internal Medicine, MercyOne Des Moines Medical Center, Des Moines, IA, USA; ^2^Division of Gastroenterology and Hepatology, University of Nebraska Medical Center, Omaha, NE, USA; ^3^Department of Gastroenterology and Hepatology, MercyOne Des Moines Medical Center, Des Moines, IA, USA

## Abstract

Esophageal squamous papillomas are rare epithelial lesions typically discovered incidentally during EGD. Their prevalence is estimated to be less than 0.01% in the general population. We present three cases of esophageal squamous papillomas identified histologically. It may be possible to identify these lesions macroscopically. One study provided a positive predictive value of 88% for squamous papilloma utilizing the triad of exophytic growth, wart-like projections, and surface vessel crossing seen on narrow band imaging during endoscopy. The etiology is unclear. Chronic mucosal irritation from GERD or esophagitis is the prevailing theory of pathogenesis, but HPV has been detected in some lesions. The malignant potential of these lesions is considered controversial. There are documented cases demonstrating complications with squamous cell carcinoma, so we recommend removal of all esophageal squamous papillomas; however, the small absolute number of cases documented in the literature makes drawing any associations or conclusions between esophageal squamous papillomas and squamous cell carcinoma difficult. Further research is needed regarding treatment and surveillance. This case series helps contribute to the small but growing literature of this rare finding.

## 1. Introduction

Esophageal squamous papillomas (ESPs) are rare epithelial lesions typically discovered incidentally during esophagogastroduodenoscopy (EGD). The occurrence in patients undergoing EGD is less than 0.45% [1] but was noted to make up 4.5% of all esophageal lesion biopsies at one medical center during a 15-year period [2], and the prevalence in the general population, inferred from autopsy, is less than 0.01% [3]. We present a case series of 3 patients found to have ESPs discovered over a one year period of approximately 600 EGDs, which is consistent with the incidence reported in the literature. Most patients are asymptomatic unless the lesion is large. The most common symptom from a squamous papilloma is dysphagia. A majority of these lesions are found in the distal one-third of the esophagus. Though rare, there are case reports demonstrating ESP complications with squamous cell carcinoma [3–6].

## 2. Cases

### 2.1. Case 1

A 53-year-old male with history of alcohol abuse presented with new-onset seizure/syncope, six painless maroon stools, and coffee-ground emesis. The patient admitted to frequent diclofenac use and ketorolac injections. He did not have a history of varices. Vital signs demonstrated tachycardia (heart rate 111 beats/min) and hypotension (79/59 mmHg). Physical exam revealed pale conjunctiva, dry mucous membranes, and maroon colored stool. He was found to be anemic with a hemoglobin of 7.9 G/dL and had an elevated INR of 1.2. He was transfused two units of packed red blood cells and was started on a pantoprazole drip.

**Figure 1:**
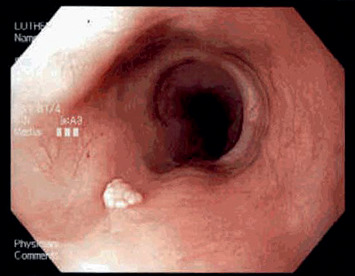
Esophageal squamous papilloma in Case 1. Esophageal squamous cell papilloma is an exophytic growth with wart-like projections on conventional endoscopy. Its whitish color is a poor differentiator from other lesions [7].

**Figure 2:**
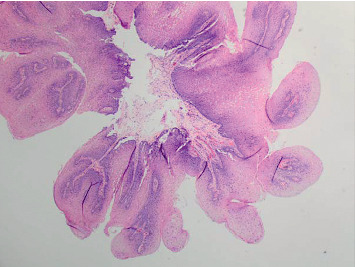
Esophageal squamous papilloma in Case 1. Lesion is diagnosed pathologically with hematoxylin and eosin stain demonstrating a fibrovascular core branching out from the lamina propria forming finger-like projections without invasion into the submucosa surrounded by marked neutrophil infiltration and covered by acanthotic squamous epithelium (H&E, *x*20) [1, 7–9].

**Figure 3:**
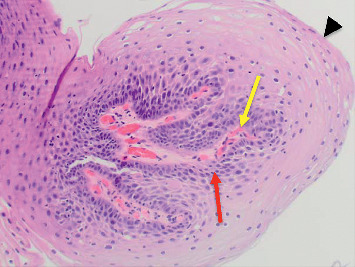
Esophageal squamous papilloma in Case 1. A fibrovascular core branching out from the lamina propria forming finger-like projections (yellow arrow) without invasion into the submucosa surrounded by marked neutrophil infiltration (red arrow) and covered by acanthotic squamous epithelium (black arrowhead) can be seen (H&E, *x*100).

Once stabilized, initial EGD revealed an island of tissue growth in the mid-esophagus and a bleeding duodenal ulcer that was injected with epinephrine, cauterized with gold probe and clipped. Biopsy of the esophageal lesion was deferred to outpatient follow-up given the severity of the patient's presenting condition. He had no further signs of gastrointestinal bleeding. Two weeks later, a repeat EGD was performed. Biopsies were taken of the stomach mucosa and mid-esophageal mass (see Figure 1). Gastric biopsy demonstrated mild chronic gastritis without dysplastic changes or *Helicobacter pylori*. Esophageal biopsy demonstrated findings consistent with squamous papilloma (see Figures 2 and 3).

### 2.2. Case 2

**Figure 4:**
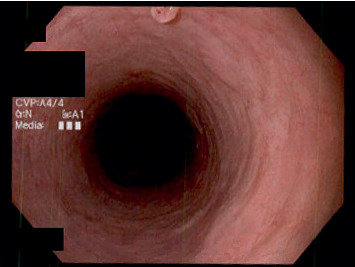
Partially visible esophageal squamous papilloma in Case 2 demonstrating exophytic growth with wart-like projections.

A 61-year-old female with history of uncontrolled diabetes mellitus type 2 complicated by gastroparesis, prior esophageal dilation, and ileostomy was being evaluated with endoscopy as outpatient for worsening dysphagia. EGD was significant for a small nodule in the proximal esophagus (see Figure 4), normal GEJ (gastroesophageal junction), mild gastritis, and normal duodenum. Gastric biopsy demonstrated mild chronic gastritis without dysplastic changes or *Helicobacter pylori*. Esophageal biopsy demonstrated findings consistent with squamous papilloma.

### 2.3. Case 3

**Figure 5:**
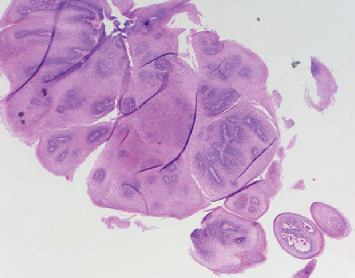
Esophageal squamous papilloma in Case 3. Note is made of fibrovascular cores branching out from the lamina propria forming finger-like projections surrounded by marked neutrophil infiltration and covered by acanthotic squamous epithelium (H&E, *x*20).

**Figure 6:**
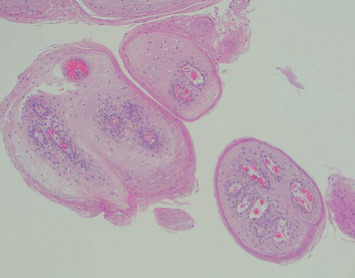
Esophageal squamous papilloma in Case 3. Several fibrovascular cores branching out from the lamina propria with finger-like projections can be seen without invasion into the submucosa. These are surrounded by marked neutrophil infiltration and covered by acanthotic squamous epithelium (H&E, *x*100).

A 54-year-old female with history of fibromyalgia, chronic opioid dependency, cyanocobalamin deficiency, vitamin D deficiency, and GERD with previously noted Los Angeles class C esophagitis was being evaluated with endoscopy as outpatient for dysphagia and abdominal pain. EGD was significant for an exophytic wart-like growth in the distal esophagus, minimally irregular *Z*-line noted at GEJ, mild gastritis without ulcers, and normal duodenum. GEJ biopsy demonstrated focal intestinal metaplasia suggestive of Barrett's esophagus without dysplastic changes. Esophageal biopsy demonstrated findings consistent with squamous papilloma (see Figures 5 and 6).

## 3. Discussion

Squamous papilloma of the esophagus is a rare lesion. Its macroscopic appearance is similar to other, less benign growths (verrucous squamous cell carcinoma, granulation tissue, or papillary leukoplakia). Other differential considerations should include fibrovascular polyp, inflammatory fibroid polyp, leiomyoma, and malignant melanoma [10]. Narrow band imaging (NBI) can further evaluate a squamous papilloma during endoscopy as microvessels within the lesion will not be dilated [11]. Based on a study of 41 esophageal polypoid lesions, the triad of exophytic growth, wart-like projections, and surface vessel crossing seen on NBI during endoscopy can provide a positive predictive value of 88.2% for squamous papilloma [7]. The study noted the whitish color of squamous papillomas does not help differentiate from other lesions.

Squamous papilloma cases usually consist of a small (<5 mm) solitary lesion that is found incidentally while evaluating a patient for abdominal pain or reflux [8, 9, 11]. Histological evaluation classically demonstrates a fibrovascular core branching out from the lamina propria forming finger-like projections without invasion into the submucosa completely surrounded by marked neutrophil infiltration and covered by acanthotic squamous epithelium [1, 7–9].

The etiology is unclear. Chronic mucosal irritation from GERD or esophagitis is the prevailing theory of pathogenesis, but HPV (human papillomavirus)—strongly associated with cervical, anal, and oropharyngeal cancers (depending on viral serotype)—has been detected in some lesions [1, 4]. A study comparing clinicopathological characteristics of Japanese patients to western countries identified 38 esophageal squamous papillomas (ESPs) in 35 patients over a period of 13 years and noted four tumors in four female patients (10.5%) tested positive for HPV subtype 6 [1]. A study evaluating HPV in 18 patients with 19 cases of ESPs in a Mexican cohort identified HPV in almost 80% of ESPs utilizing amplified chromogenic in situ hybridization or PCR though all cases exhibited low expression of cell-cycle markers [9].

There have been several other studies with HPV-positive ESP results ranging from 0 to 64% and with different esophageal lesion location distribution curves indicating that differences in detection methods or possibly geographical/environmental exposure factors may play a role in prevalence [1]. Our three cases did not test positive for HPV.

Complications with squamous cell carcinoma (SCC) have been reported [3–6]; however, the small absolute number of cases makes drawing any associations or conclusions between ESP and SCC difficult [12]. Review of the literature seems to suggest malignant potential appears highest in cases where the patient is symptomatic and has multiple lesions (papillomatosis), or a particularly large lesion [5, 6].

The gold standard for evaluation is biopsy. There are various proposed therapies for squamous papillomas including forceps biopsy resection, cautery, radiofrequency ablation, mucosectomy for larger lesions, and potentially esophagectomy if presentation is extreme and does not respond to more conservative therapy [4, 5, 11, 13, 14]. Future research is needed regarding treatment and surveillance for these lesions. Though rare, there are documented cases demonstrating malignant potential, so we recommend removal of esophageal squamous papillomas.

## Data Availability

There is no database for this case series. All important findings/data are available within the text.
